# A review of alterations to the brain during spaceflight and the potential relevance to crew in long-duration space exploration

**DOI:** 10.1038/s41526-021-00133-z

**Published:** 2021-02-16

**Authors:** Meaghan Roy-O’Reilly, Ajitkumar Mulavara, Thomas Williams

**Affiliations:** 1 Department of Neurology, University of Texas Health Science Center, Houston, TX USA; 2 Department of Medicine, Stanford University Medical Center, Stanford, CA USA; 3 KBR, Houston, TX USA; 4 National Aeronautics and Space Administration, Johnson Space Center, Houston, TX USA

**Keywords:** Neurology, Medical research, Neuroscience

## Abstract

During spaceflight, the central nervous system (CNS) is exposed to a complex array of environmental stressors. However, the effects of long-duration spaceflight on the CNS and the resulting impact to crew health and operational performance remain largely unknown. In this review, we summarize the current knowledge regarding spaceflight-associated changes to the brain as measured by magnetic resonance imaging, particularly as they relate to mission duration. Numerous studies have reported macrostructural changes to the brain after spaceflight, including alterations in brain position, tissue volumes and cerebrospinal fluid distribution and dynamics. Changes in brain tissue microstructure and connectivity were also described, involving regions related to vestibular, cerebellar, visual, motor, somatosensory and cognitive function. Several alterations were also associated with exposure to analogs of spaceflight, providing evidence that brain changes likely result from cumulative exposure to multiple independent environmental stressors. Whereas several studies noted that changes to the brain become more pronounced with increasing mission duration, it remains unclear if these changes represent compensatory phenomena or maladaptive dysregulations. Future work is needed to understand how spaceflight-associated changes to the brain affect crew health and performance, with the goal of developing comprehensive monitoring and countermeasure strategies for future long-duration space exploration.

## Background

Recent advances and renewed investment in human spaceflight have accelerated the timeline for long-duration space exploration missions, including crewed missions to Mars. The success of these endeavors is contingent on our ability to monitor and maintain human health and performance during the mission. Maintaining the integrity of the central nervous system (CNS) and the brain during long-duration space exploration is a high priority, because high-level sensorimotor and cognitive processes are essential to many mission critical tasks.

Decrements in operational performance have been reported throughout the history of spaceflight^1^. Crewmembers of the short-duration Apollo era missions reported altered driving performance on lunar excursions^2^, and during the first 100 Space Shuttle Missions 20% of orbiter landings fell outside acceptable limits^3^. In-flight performance decrements have been noted during missions aboard Mir and the International Space Station (ISS), with reports of several close calls and one collision between a vehicle and space station components^4^. Although current and future generations of NASA spacecraft have been designed for autonomous flight, the crew must be capable of manually operating the vehicle in case the automatic control fails^5^.

Previous studies have demonstrated changes across multiple neurologic domains after spaceflight including changes to sensation, movement, coordination, and cognition^5^. In particular, sensorimotor dysfunction has been implicated as a major potential cause of performance decrements during spaceflight^6,7^. Sensorimotor deficits reported during and after spaceflight include impaired gaze control, reduced fine motor control, spatial disorientation, impaired coordination, postural ataxia, and loss of motor efference^4^. Of note, whereas these sensorimotor changes are greatest immediately after gravitational transitions, the extent and duration of some of these alterations have been associated with increased mission length^4,8,9^. As an example, poorer landing accuracy in Space Shuttle pilots was associated with longer mission duration and greater vestibular dysfunction, as determined by postflight assessment^1,7^.

**Fig. 1:**
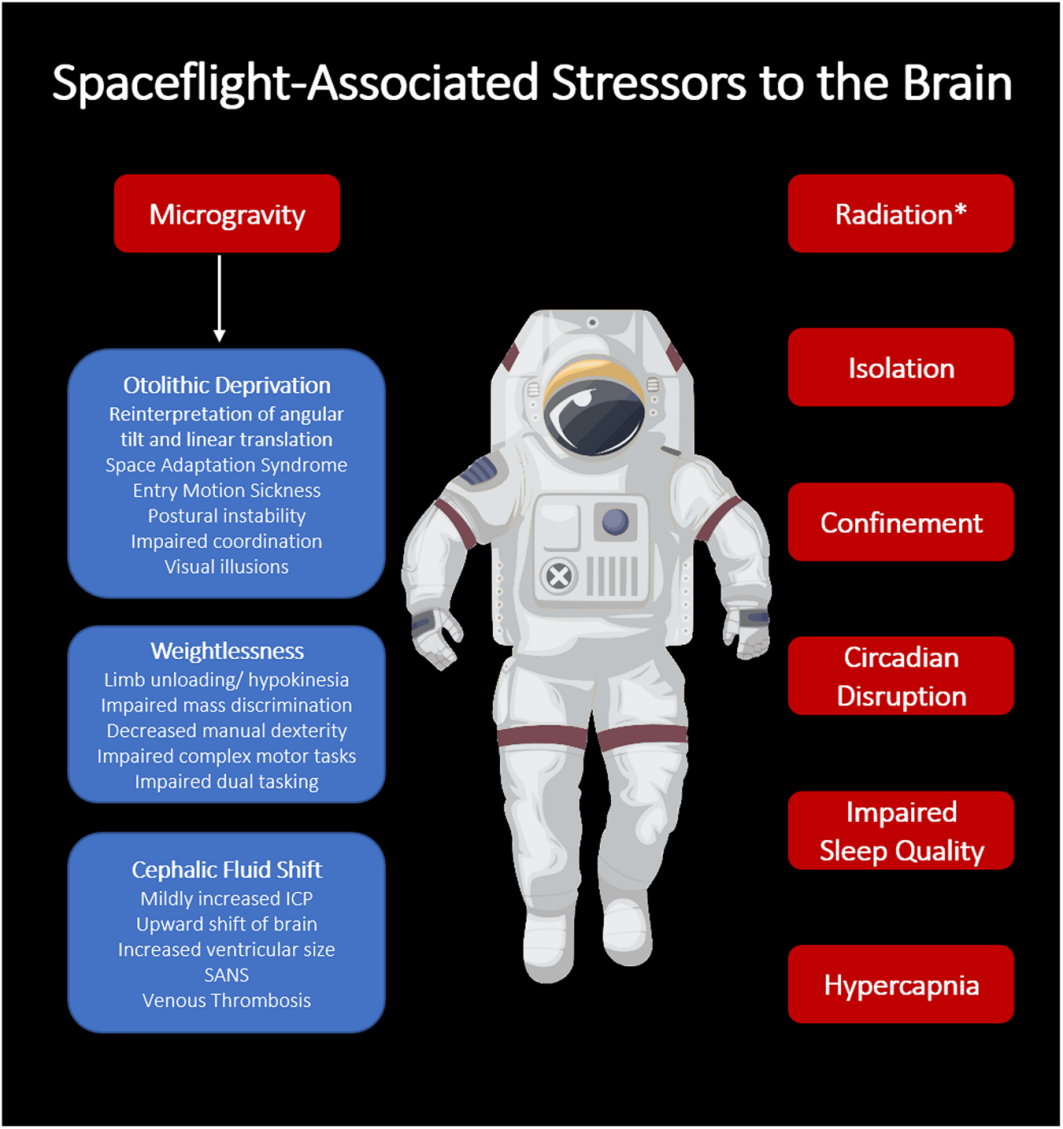
Summary of environment stressors with potential to impact the brain during spaceflight. ICP = Intracranial Pressure. SANS = Spaceflight-Associated Neuro-Ocular Syndrome. *Radiation in this case refers to Deep Space Radiation, a hypothesized but as of yet unproven risk of deep space exploration, rather than the radiation experienced in Low-Earth Orbit (LEO) missions.

Space is a unique environment, making the investigation of spaceflight-associated changes to the brain a complex task. Microgravity itself is believed to affect the brain via multiple mechanisms, including vestibular deprivation, weightlessness, and cephalic fluid shift^10^. However, a growing body of evidence suggests that other spaceflight-associated factors may also impact the brain, including space radiation, isolation and confinement, circadian disruption, and chronic hypercapnia (Fig. 1)^1^. Therefore, it is likely that any gross changes observed in operator performance result from the combinatorial and cumulative effects of multiple spaceflight-associated stressors on individual brain regions^5,11^.

Magnetic resonance imaging (MRI) is a high-resolution neuroimaging technique and powerful supplementary tool that allows detailed assessment of the structure and functional connectivity of the brain. Past reviews of spaceflight-associated changes to the brain have highlighted results from neuroimaging studies conducted in animal models and in humans who are exposed to analogs of spaceflight^12,13^. However, recently published MRI studies have now demonstrated changes after spaceflight itself, including changes in brain position, tissue volume, ventricular volume, cerebrospinal fluid (CSF) distribution and dynamics, tissue microstructure, and functional connectivity^13–21^. In this review, we aim to summarize current knowledge regarding neuroimaging-detected changes to the structure and connectivity of the brain after spaceflight, particularly as they relate to mission duration.

## Review of current knowledge regarding spaceflight-associated changes in the Structure and function of the brain

To date, multiple MRI modalities have been used to assess the structure and function of the brains of crewmembers before and after spaceflight at both the anatomical level and the microstructural level (Supplementary Table 1)^13,14,16–22^. Several studies have also used functional MRI (fMRI) to examine the connectivity within or between different brain regions during rest or activated states^15,23^. A summary of the findings from currently available MRI-based studies examining changes to the brain after spaceflight are summarized in Table 1.

**Table 1:** Summary of MRI findings from studies conducted in US astronauts and Russian cosmonauts pre and post spaceflight and potential clinical correlates where applicable.

Study	Population	Imaging protocol	Main findings
Demertzi et al. ^15^	*n* = 1 LD. Pre and post flight scans	fMRI (3 T): -Resting state connectivity -Active mental imagery connectivity	- Reduced intrinsic connectivity in R insula and ventral posterior cingulate cortex; reduced connectivity between R motor cortex and L cerebellum (resting state fMRI) -Increased activation of the supplementary motor area (active imagery fMRI)
Koppelmans et al.^13^	*n* = 27, 13 SD and 14 LD. Pre- and post flight scans	T2-weighted MRI (3 T) -Gray matter volume	-Widespread GM decreases in frontal and temporal poles -Focal GM decreases in R inferior frontal gyrus, R frontal pole, L temporal lobe, L insular cortex. Changes greater in LD crew.-Focal GM increases in medial primary somatosensory and motor cortices
Roberts et al.^18^	*n* = 34, 16 SD and 18 LD Pre- and post flight scans	T2-weighted MRI (3 T) -Static structure changes T1-weightedl MRI (3 T), cine-clips -Brain position and CSF spaces	-Narrowing of CSF spaces, increase in width of 3^rd^ ventricle, upward movement of cerebellar tonsils. Changes greater in LD crew.-Upward shift of brain and brain stem, narrowing of CSF spaces at the vertex, rotation of the cerebral aqueduct, stretching of the pituitary stalk. Occurred at higher rates in LD crew.
Alperin et al.^14^	*n* = 17, 7 SD and 10 LD Pre- and post flight scans 1 month follow up scan	T1-weighted MRI (3 T) -Ventricular volumes T2-weighted FLAIR MRI (3 T) -WMH	-Increased periventricular WMH in LD group only. -No significant increase in deep WMH -Increase in total ventricular volume, significantly positively associated with periventricular WMH -Partial reversal of observed increases at 1-month follow-up scan
Van Ombergen et al.^20^	*n* = 10 LD. Pre- and post flight scans 7-month follow-up scan	T1-weighted MRI (3 T) -Brain tissue and CSF volumes	-GM volumes decreased in orbitofrontal and temporal cortexes, largely recovered by 7-month follow-up scan. -Cerebral WM volumes globally reduced at 7-month follow-up scan compared to early postflight scan. -CSF spaces and ventricles increased in volume (maximal at third ventricle), decreased CSF volume below the vertex. -At 7-month follow-up, CSF volume in the ventricles partially returned to preflight values, while CSF volume in subarachnoid space increased.
Roberts et al.^19^	*n* = 19, 7 SD and 12 LD Pre- and post flight scans	T1-weighted MRI (3 T) -Brain tissue and CSF volumes	-Increase in total ventricular volume in LD crew. Significant increase in lateral ventricles and third ventricle, but not in fourth ventricle. Change in ventricular volume associated with increasing flight duration and decreasing crewmember age. -Crowding of parenchyma at the vertex (supplementary motor, premotor, primary sensorimotor regions) and displacement of brain tissue surrounding the ventricles seen in LD crew only. -Clinical Correlate: Change in L caudate associated with poorer postural control, change in R lower extremity primary motor area/midcingulate associated with longer time on seated egress and walk test. Reduced accuracy on WinSCAT code substation learning subtest, faster reaction times associated with smaller changes in volume of bilateral optic radiations and splenium
Van Ombergen et al. 2019^21^	*n* = 11, LD Pre- and post flight scans, 7-month follow-up	T1- weighted MRI (3 T) -CSF volumes	-Increased lateral ventricle, third ventricle and total ventricular volume after LD spaceflight, with residual baseline increases at 7 months. No significant change in fourth ventricle. -Ventricular volume increases associated with increasing mission duration
Riascos et al.^17^	*n* = 19, 10 SD and 9 LD Pre- and post flight scans	T1-weighted MRI (3 T) -Brain tissue volume Diffusion-weighted MRI (3 T) -WM microstructure, GM diffusivity	-Trend for cortical thinning of R occipital lobe; reduced volume of L. thalamus; increased lateral ventricular volume. -WM changes in R posterior thalamic radiations, trend towards greater effects in LD crew.
Lee et al.^16^	*n* = 15, 7 SD and 8 LD Pre- and post flight scans	Diffusion-weighted MRI (3 T) -WM microstructure, GM diffusivity	-Increased FW volume in frontal, temporal and occipital lobes after spaceflight, with decreased FW volume at the vertex. -WM changes in R. SLF, ILF, IFOF, CST, ICP, MCP and white matter structures underlying the precentral and postcentral gyrus, supramarginal gyrus and angular gyrus. -Greater WM organization postflight in cerebellar white matter in LD crew compared to SD crew -Clinical Correlate: Microstructural changes in the SLF were greater in individuals with greater decreases in balance control
Pechenkova et al.^23^	*n* = 11 LD, compared to HC Pre- and post flight scans	fMRI (3 T): -Resting state connectivity -Plantar stimulation paradigm	-Stimulation-specific increase in connectivity of R posterior supramarginal gyrus with the rest of the brain -Increased connectivity between R and L posterior insula; decreased connectivity between posterior cerebellum and primary visual cortex; decreased connectivity between anterior cerebellum and R parietal cortex -Connectivity modifications at vestibular nuclei, R parietal cortex, anterior cerebellar network, R posterior insula and L posterior insula -Clinical Correlate: Space motion sickness severity associated with connectivity between R posterior supramarginal gyrus and L insular region
Kramer et al.^22^	*n* = 11, LD Pre- and post-flight scans 1, 3, 9, 12-month follow-up scans	T1- and T2 weighted MRI (3 T) -Brain tissue and CSF volumes -Pituitary evaluation T1-weighted MRI (3 T), cine-clips -Quantitative CSF Flow	-Expansion of total brain and CSF volumes after LD spaceflight, with persistent elevation 1 year after spaceflight. Largely driven by global WM volume and lateral ventricular volume increases -Increased aqueductal stroke volume and CSF peak-to-peak velocity magnitude -Pituitary depression seen in 6/11 crewmembers
Hupfield et al.^24^	*n* = 12, LD (6-month, *n* = 10, 12-month n = 2), compared to HC Pre and post-flight scans 6 month follow-up scans	T1-weighted MRI (3 T) -Brain tissue and CSF volumes Diffusion-weighted MRI (3 T) -FW shifts	-Significantly enlarged ventricular volumes in all 6-month mission astronauts, and 1 of 2 12-month mission astronauts compared to control. Partial resolution of increased volumes seen 6-months post-flight in 50% of subjects -Increased GM volume and cortical thickness in the SMA, pre- and postcentral gyri in the 6-month mission astronauts and 1 of 2 12-month mission astronauts, largely recovered on 6-month follow-up scan
Jillings et al.^25^	*N* = 11, LD, compared to HC Pre and post-flight scans 7 month follow-up scans	Diffusion-weighted MRI (3 T) -Multi-tissue spherical deconvolution for GM, WM and CSF volumes	-GM changes driven by local volume shifts rather than tissue loss, largely reversed at 7-month follow up -WM volume increases in the cerebellum, CST, PMC -GM volume increases in the basal ganglia -Larger visual acuity decreases post-flight associated with greater ventricular expansion

*CSF* cerebrospinal fluid, *CST* corticospinal tract, *FW* free water, *GM* gray matter, *HC* healthy control, *ICP* inferior cerebellar peduncle, *IFOF* inferior fronto-occipital fasiculus, *ILF* inferior longitudinal fasiculus, *L* left, *LD* long duration, *PMC* primary motor cortex, *MCP* middle cerebellar peduncle, *R* right, *SD* short duration, *SLF* superior longitudinal fasiculus, *SMA* supplementary motor area, *WMH* white matter hyperintensities.

### Changes to cerebrospinal fluid circulation after spaceflight

Neuroimaging studies have detected numerous spaceflight-associated changes in the positioning and macrostructure of the brain, including alterations to the ventricular system, which contains cerebrospinal fluid (CSF)^14,17–22^.

In 2017, Roberts et al. reported widespread structural change within the brain after spaceflight, including narrowing of the central sulcus, upward shift of the brain, twisting of the cerebral aqueduct, and increased ventricular volumes^18^. A follow-up study demonstrated an increase in total ventricular volumes in crewmembers of long-duration ISS missions (10.7%) that was absent in crewmembers of short-duration Space Shuttle missions (0%)^19^. Within the cohort of long-duration fliers, a continuous linear association (*r* = 0.72) was seen between increasing mission duration and greater total ventricular volume^19^. Of note, volume increases were primarily observed in the left lateral ventricle (LLV, 17.1%), right lateral ventricle (RLV, 15.2%), and third ventricle (3 V,15.4%) with relative sparing of the fourth ventricle (4 V, −0.83%)^19^. Van Ombergen et al. reported similar findings, with significant increases in the volume of the LV (13.3%) and 3 V (10.4%) after long-duration spaceflight and minimal change in the 4 V^21^. On 7-month follow up-scans, the ventricular volumes had decreased from the initial postflight measurements but still remained significantly elevated from preflight measurements^20,21^. Kramer et al. reported increased ventricular volumes in astronauts 1 day after they returned from long-duration spaceflight, which were persistently elevated at 12 months post-flight^22^.

A recent study by Hupfield et al. examined fluid shifts and ventricular volume changes within a group of long-duration ISS astronauts^24^. The authors found greater fluid shifts in astronauts following 12 months in space compared to 6 months, which returned to baseline by 6 months post-flight^24^. Ventricular volumes were significantly increased in all astronauts in the 6-month mission group (17 ± 12% LLV, 24 ± 6% RLV). Interestingly, 1 of 2 subjects studied following a 12-month mission demonstrated significantly increased ventricle volumes (25% LLV, 23% RLV) while the second subject showed smaller percent increases (2% LLV, 3% RLV), potentially due to larger baseline ventricular size^24^. On 6-month follow-up scan, approximately half of the 6-month mission astronauts demonstrated post-flight resolution of ventricular volume changes, while the other subset showed continued ventricular enlargement post-flight^24^. Subsequent analysis demonstrated that LV volumes were correlated with number of post flight days, number of previous missions and less time between missions; no effect of age was seen on pre-flight ventricular volume^24^.

### Structural gray and white matter alterations after spaceflight

The brain itself is composed of two primary tissue types, known as gray matter (GM) and white matter (WM). GM, composed of neuron cell bodies and glial cells, is responsible for modifying and collecting information transmitted from local neurons and from distant regions via bundles of long, myelinated axons known as WM tracts.

Several studies have demonstrated no significant change in global GM or WM volume following spaceflight in astronauts or cosmonauts^13,14,19,20^. However, Kramer et al. recently reported a significant increase in the global WM volume of long-duration astronauts immediately after spaceflight (5.5%) that persisted out to 12 months (4.6%), with no significant changes to the global GM on post-flight scans^22^. Kramer et al. also reported increased summated mean brain and CSF volumes post-flight, which were not seen in previous studies^13,20,22^. The difference in these findings may be attributable to alternative volumetric quantification methods; total pre-flight WM volume in prior studies averaged 540–580 ml, while in the recent Kramer study reported pre-flight WM volumes between 417–470 ml. This discrepancy highlights an important point; a standardized method of quantification for neuroimaging in spaceflight is key to improved accuracy and reproducibility of future studies.

Given the high regional specificity of neurologic functions, examining focal alterations within brain tissue, rather than global tissue shifts, may yield greater insight into the neurologic regions affected during long-duration spaceflight. Several studies have reported focal GM changes in the brain following spaceflight. Decreases in GM volume of the orbitofrontal and temporal poles have been reported in both astronauts and cosmonauts following spaceflight^13,20^. Koppelmans et al. has previously noted increased GM in sensorimotor and motor areas of the brain^13^, a finding echoed by recent work by Hupfield et al. showing increased GM in the SMA, pre-central and post-central gyrus^24^. It is important to note that the majority of these GM alterations overlapped significantly with alterations in CSF and FW, as well as shifting of brain position in the skull. In addition, GM volume changes were largely resolved approximately 6-months post-flight^20,24^.

Jillings et al. has recently published an MRI study in cosmonauts utilizing a technique called multi-tissue spherical deconvolution with the goal of differentiating GM changes driven by local volume shifts (CSF, brain positioning) versus genuine tissue loss such as that seen in neurodegeneration^25^. They demonstrated that the vast majority of GM changes were due to volume shifts; these GM changes were largely reversed on 7-month follow up, in agreement with previous studies^25^. Interestingly, by their method, a net gain of GM tissue was noted in the basal ganglia, an area involved in voluntary movement^25^. In addition, Jillings et al. noted increased WM volume in the cerebellum, corticospinal tract and primary motor cortex in cosmonauts immediately following spaceflight, which were partially resolved on 7 month follow-up scan. Taken together, these results may suggest that spaceflight induces WM volume increases in the motor and coordination regions of the brain, which partially resolves upon return to earth^25^.

Several studies have also utilized diffusion-weighted MRI to examine the microstructure of WM by fractional anisotropy. Lee et al.^16^ have shown focal changes in WM microstructure after spaceflight within multiple sensory regions, including regions important to vestibular and proprioceptive processing^16^. These areas included the inferior cerebellar peduncle, which transmits information between the vestibular receptors and the cerebellum, and the right inferior and posterior parietal lobe, which integrate vestibular and proprioceptive information and play an important role in perception of the spatial representation of the body and upright perception^16^. Lee et al. also noted WM changes in regions related to vision and visual processing, including the superior longitudinal fasiculus (SLF), which plays an important role in visuospatial processing, visual attention, and visuomotor control^16,26^. Interestingly, greater changes in the SLF in astronauts was found to be correlated with larger postflight balance disruptions^16^. WM changes were also noted in the inferior longitudinal fasiculus (ILF) and inferior fronto-occipital fasiculus (IFOF), which run along the lateral ventricle in close proximity to the optic regions and are believed to play a role in visual recognition (object, facial, emotional), visual processing, visually-guided decision-making and language comphrension^2,16^.

Riascos et al. recently reported spaceflight-associated WM changes within areas associated with vision and visual processing^17^. WM was altered in the posterior thalamic radiations, which contain the optic radiations and help carry visual information between the thalamus and occipital cortex. Interestingly, investigators also noted GM changes to both the thalamus and occipital cortex were also noted, which were hypothesized by the authors to occur secondary to fluid accumulation in the visual pathway or to the downstream effects of changes within the optic nerve itself^17^.

Alteration of the microstructure of WM tracts involved in motor function was also described by Lee et al., including decreased organization in the corticospinal tract and the WM underlying the primary motor cortex, theorized to occur secondary to the decreased use of the lower limbs in space^16^. WM changes were also detected in the middle cerebellar peduncle, a region that contains the cortico-ponto-cerebellar tract that facilitates initiation, timing and planning of movement^16,27^. Interestingly, astronauts had greater baseline WM organization in the corticospinal tract and cerebellar peduncles compared to control subjects^16^. Because all astronauts included in this study had previous spaceflight exposure, this may demonstrate an adaptive change in motor and balance control induced by spaceflight, and/or induced by the spaceflight simulations and other countermeasures used to prepare crewmembers for spaceflight exposure^16^. In addition, Lee et al. reported that that crewmembers of long-duration missions had greater postflight WM organization in the cerebellar white matter than did crewmembers of short-duration missions, indicating this adaptation may accumulate with increasing time in space^16^.

### Functional brain changes after spaceflight

The first study of altered brain connectivity after spaceflight was conducted by Demertzi et al. in a single Russian cosmonaut^15^. The investigators found decreased connectivity within the right insula, which is involved in vestibular processing and cognitive control, and altered connectivity between the motor cortex and cerebellum^15^. A second, larger study by Pechenkova et al. also demonstrated that spaceflight decreased functional connectivity of the cerebellum to regions with proprioception, visual, motor, and somatosensory functions^23^. The cerebellum is important for coordination and fine-motor control, and is believed to play a significant role in sensorimotor adaptation to microgravity^28,29^. Pechenkova et al. found decreased connectivity between the vestibular nuclei and sensory and motor regions of the brain after flight, which is believed to result from central adaptation that down-regulates vestibular input during spaceflight, reducing sensory conflict and helping to ameliorate space motion sickness^23,30^.

## Comparison of spaceflight-related CNS alterations to those seen in terrestrial analogs

Spaceflight analogs are useful for studying the individual contributions of spaceflight-associated stressors on the CNS. Similarly, shared features of spaceflight-associated neurologic decrements and features of terrestrial human disease may help identify biomarkers and inform future countermeasure strategies.

Head-down bed rest (HDBR) at a 6^0^ tilt likely represents one of our greatest assets in assessing the influence of spaceflight-analog conditions on the brain, as it exposes subjects to cephalic fluid shift, hypokinesia, unloading of the axial body and environmental depriviation^31^. Several common neuroimaging changes are induced in HDBR and in spaceflight, including rotation of the brain within the skull, crowding of tissue at the vertex, alterations in free water, and decreases in front-orbital and temporal GM^32–35^.

HDBR and spaceflight also induce similar changes in brain connectivity, including altered connectivity of motor, somatosensory and vestibular areas of the brain^13,36^. In addition, performance of sensorimotor and spatial working memory tasks changes, balance and locomotor function declines, neural efficiency during vestibular stimulation decreases, and neurocognitive reserve during dual tasking decreases during spaceflight and HDBR^13,36–38^. These data suggest that alterations in the thalamus, parietal cortex, and motor and sensory cortices with their associated declines in sensorimotor, balance, and dual task performance may be related to altered mobility and axial body unloading.

Spaceflight analogs have been able to recapitulate some of the findings from spaceflight studies, including changes in connectivity, GM volumes, CSF dynamics and ventricular volumes. However, evidence of WM changes during HDBR remain equivocal. Koppelmans et al. reported no WM changes after 70 days of HDBR, whereas Li et al. reported widespread alterations in WM after 30 days of HDBR without adjustment for multiple comparisons in the statistical tests used. Similarly, studies have demonstrated mixed results regarding ventricular volume changes after HDBR^32–35,39^. Interestingly, recent work has demonstrated that strict adherence to HDBR reproduces SANS-like pathology in an analog setting, suggesting that these equivocal results may be the result of methodological differences in study design and protocol adherence, and that hypercapnia may play a role in the development of SANS pathology^40^. However, no HDBR study to date has been able to reproduce the full spectrum of ocular changes seen in SANS, as the globe flattening and visual acuity changes seen in long-duration microgravity were absent in terrestrial studies.

Interestingly, the subjects who developed SANS-like pathology in the aforementioned HDBR experiment by Laurie et al. were also exposed to chronic mild hypercapnia as part of the study design (HDBR with CO_2_)^40^. Previous research suggests that chronic hypercapnia itself may have detrimental effects on the CNS^41^. Crewmembers on the ISS are chronically exposed to levels of CO_2_ that are 10-fold higher than those experienced on Earth, and some reports document increased sensitivity to elevated CO_2_ during spaceflight^42^. Kramer et al. demonstrated that combined HDBR and CO_2_ results in increased ventricular volumes and altered CSF hydrodynamics^39^. Subsequent work by the authors demonstrated that CSF hydrodynamics were altered immediately after spaceflight itself, in conjunction with increased postflight ventricular volumes^22^. Elevations in arterial CO_2_ can decrease CSF absorption by 50% in an animal model, offering one potential mechanism for the additive effect of HDBR and CO_2_ on the ventricular system^43^. Further experiments are required to determine if both the cephalic fluid shift during long-term HDBR and the elevated CO_2_ levels are both necessary and sufficient for increased ventricular volume.

Several recent studies have also explored HDBR with CO_2_ as an enhanced model for sensorimotor re-weighting during spaceflight^44–46^. A recent study of HDBR in an elevated CO_2_ atmosphere demonstrated altered activation of the brain during vestibular stimulation than during HDBR alone, indicating that increased CO_2_ may alter vestibular processing and compensation^44^. HDBR with CO_2_ has also been associated with greater decreases in activation of the left inferior temporal gyrus and right hippocampus compared to HDBR alone, regions that are involved in visual memory, cognition and spatial working memory^46^. Fascinatingly, a recent fMRI study demonstrated increased functional connectivity between visual, vestibular and motor brain regions during HDBR with CO_2_; while decreases in functional connectivity were seen between somatosensory, cognitive, motor, vestibular and multimodal integration regions^45^. Subjects with increased connectivity between motor and visual regions demonstrated decreased consistency in visual perception and greater slowing on Functional Mobility testing following bed rest^45^. As this study did not involve concurrent control groups for HDBR or CO_2_ alone, further study is required to clarify the relative contributions of each experimental factor to sensorimotor reweighting in a spaceflight analog setting^45^.

In addition to physiological stressors, it is also important to consider how long-term isolation and confinement affect the brain. Images obtained during the MARS500 project showed alterations in WM microstructure in the right temporoparietal junction (TPJ) of subjects after they spent 520 days in isolation; changes were thought to occur secondary to exposure to reduced space and low environmental enrichment^47^. It is possible that alterations to the TPJ during long-term isolation and confinement on a crewed space mission may impair the ability of the crew to adapt to the highly novel, complex environment of Mars. Neuroimaging studies conducted in subjects after a 14-month Antarctic expedition demonstrated reduced GM volumes in the orbitofrontal cortex, prefrontal cortex, and hippocampus, indicating these regions may also be susceptible to change during environmental deprivation^48^. Social isolation and environmental deprivation can reduce hippocampal neurogenesis in animal models, an effect that may negatively affect memory and social interaction between crewmembers^49^. Interestingly, several studies have demonstrated that these brain regions are altered in spaceflight analogs and after spaceflight itself^16,20,34,50^.

## Potential relevance of CNS changes to crew health and performance

Many spaceflight-related CNS alterations likely represent beneficial adaptations to the novel environment of spaceflight. However, given both the unprecedented duration and magnitude of the spaceflight-related stressors, it is important to examine whether changes within the CNS have the potential to become pathologically dysregulated during future long-duration space exploration.

### Potential consequences of spaceflight-associated sensorimotor and cognitive alterations

In addition to alterations to the peripheral input and output pathways, the sensorimotor changes that occur during spaceflight may result in extensive central adaptation extending to the cortical level. For example, the neurovestibular system includes peripheral sensory organs that relay visual, proprioceptive and vestibular information to the brain stem for processing and integration with signals from the cerebellum and cerebral cortex. It has been suggested that long-term deprivation of vestibular senses may result in adaptation of the entire thalamocortical vestibular system, including areas involved in cognitive functions such as sensory integration^51^. Studies that support this theory have demonstrated dual-tasking of sensorimotor and cognitive behaviors is impaired during both short and long-duration space missions^6,52–54^. However, data from majority of studies examining changes in cognitive performance during long-duration spaceflight and analog environments have been largely inconclusive^55^. A novel neurocognitive assessment tool, *Cognition*, was developed to address some of the gaps in the current cognitive test battery used to assess US astronauts^56^. Recently published work from the NASA Twins Study demonstrated that one subject had significant decrements in performance as determined by the *Cognition* battery after 340 days of spaceflight as compared to both his own baseline and that of his twin, who served as a terrestrial control^57^. A longitudinal study is ongoing to examine the effects of long-duration spaceflight on neurocognitive performance paired with neuroimaging results from anatomical and functional MRI^58^.

### Potential consequences of spaceflight-associated CSF redistribution and ventricular alterations

In response to reports of an association between white matter hyperintensities (WMH) and cognitive alterations in high-altitude pilots, Alperin et al. demonstrated that increased WMH were also seen in astronauts after long-duration flight, and these increases were associated with increased ventricular volumes. However, WMH in astronauts were restricted to the periventricular region and had partially reversed by one month after return to Earth^14^. Smooth WMH, as seen by Alperin et al. in crewmembers after spaceflight, have previously been associated with subependymal gliosis and trans-ependymal CSF diapedesis, and are not believed to be ischemic in nature^14,59^. Given the association between increased ventricular volumes and the smooth periventricular WMH and the absent deep WMH after spaceflight, it is possible that these alterations may represent trans-ependymal CSF diapedesis that are at least partially reversible after spaceflight.^14,21^ Alperin et al. conclude that the absence of deep WMH in astronauts suggest that cognitive changes after spaceflight are likely unrelated to WMH burden, unlike the hypoxia-related deep WMH pattern seen in high-altitude pilots^14^.

Cephalic fluid shift and CSF redistribution have also been implicated in the etiology of spaceflight-associated neuro-ocular syndrome (SANS), a disorder characterized by ocular changes in crewmembers during long-duration spaceflight, including optic disk edema, globe flattening, choroidal folds, cotton wool spots and a hyperopic refractive change. Van Ombergen et al. noted postflight increases in CSF spaces near the optic nerve^20,60^, and Roberts et al. found that crewmembers who developed SANS had smaller changes in ventricular volume than crewmembers without SANS symptoms^19^. While preliminary, this data supports one existing theory that CSF entry into the optic nerve may contribute to SANS, and may be more likely in crewmembers who have insufficient ventricular expansion^61,62^. However, it must be noted that a more comprehensive understanding of SANS pathogenesis is still developing, and a single driving mechanism has yet to be fully proven^63^. For a comprehensive review of SANS pathophysiology and current research, we direct readers to a recent review by Lee et al.^63^.

Similarly, the pathogenesis and clinical implications of spaceflight-associated ventricular changes to the brain itself remain largely unknown. It is important to note that several hypotheses exist regarding the clinical implications of spaceflight-associated ventricular changes, and that the topic remains a highly controversial one within the field. Recently, several investigators have hypothesized that the effects of spaceflight on brain structure are similar to those seen in normal pressure hydrocephalus (NPH)^19,22^, a condition characterized by an enlargement of cerebral ventricles without increased intracranial pressure, resulting in progressive gait apraxia, cognitive impairment and late-stage urinary incontinence^13,14,19^. Subsequent work reported an association between expanded ventricular volume and periventricular tissue displacement, with a trend towards poorer postural control in crewmembers with greater changes in ventricular volume^19^. Crowding at the vertex, enlarged ventricles, periventricular WMH, and a similar pattern of decreased GM volumes occur after both long-duration spaceflight and in NPH^13,14,19^. Interestingly, several tracts identified in our review of postflight neuroimaging have also been reported to be affected in NPH, including the IFOF, thalamic radiations and the ILF^64^. In contrast, Williams et al. posit that the enlargement of the ventricles in crewmembers does not meet the clinical criteria for hydrocephalus, and that the comparison to NPH has limited utility given the absence of NPH-like symptoms in crewmembers after flight^65^. The controversial nature of this topic represents an important gap in knowledge, and underscores the importance of further research into the clinical and operational significance of ventricular volume changes after spaceflight.

### The relationship between spaceflight-associated brain changes and mission duration

Although the exact pathogenesis and clinical impact of spaceflight-associated alterations in brain structure and function remains unknown, several studies reviewed here provide evidence for an association between mission duration and the extent of changes as determined by neuroimaging. Greater rates of brain structure changes were recorded after long-duration missions, including greater increases in CSF space narrowing, upward brain shift, and pituitary stalk stretching^18^. Increases in total ventricular volume were greater in long-duration fliers than in short-duration fliers, and within the cohort of long-duration fliers, increases in ventricular volume were positively associated with mission duration^19,21^. Potential sequelae of ventricular expansion, including periventricular WMH and displacement of tissue surrounding the ventricles, were observed only in long-duration fliers^14,19^. Given that these studies demonstrate only partial resolution of these changes 6 to 12-months post flight, and that elevated pre-flight ventricular volumes are seen in astronauts with cumulatively higher mission numbers and flight days, the alterations of ventricular volumes in spaceflight appear to result in sustained ventricular expansion beyond the rate expected with normal aging^20,22,24^. Importantly, prolonged spaceflight exposure may also have operationally beneficial adaptations; Lee et al. noted that crewmembers of long-duration missions had greater WM coherence in the corticospinal tract and cerebellar peduncles, a finding they postulated may reflect motor adaptation secondary to repeated gravitational exposure^16^.

Taken together, these results indicate that some brain alterations become more pronounced with increasing mission duration. Current long-duration neuroimaging studies have recorded minor brain alterations after an average of 6 months of spaceflight; further progression of these symptoms during longer periods of spaceflight exposure, such as during a multi-year interplanetary expedition, could significantly affect a mission. Further study is required to determine whether individual alterations represent adaptive or maladaptive processes, based on their impact to crew health and operational performance during a given mission phase.

### Current limitations of neuro-imaging studies in spaceflight subjects

It is important to note that the interpretation of virtually all spaceflight neuroimaging studies is limited by significant study limitations reflective of the exceedingly small n of their astronaut and cosmonaut subjects. As MRI can only be performed terrestrially, there remains no current ability for high resolution neuro-imaging in flight, limiting our knowledge of spaceflight-associated brain alterations to those with residual effects that last post-landing. In addition, many studies are limited by the time delay between pre and post-flight MRI assessments; changes during this time could also be reflective of training effects during the intensive preparation crewmembers perform leading up to their flight^19,22,33^. In addition, the timing of the scans between subjects was not uniform due to the retrospective nature of the studies, and much of the MRI data available was of low-resolution, increasing the risk of measurement variance and volumetric error^14,16,33^. Lack of an age or sex matched longitudinal health control group in many of the studies also limits the ability to interpret which brain changes are directly related to spaceflight exposure vs. baseline or training related alterations that may be inherently present in the elite subset of the population our crewmembers represent^17,22^. Notably, the data on female astronauts or sex differences in neuroimaging after spaceflight is very sparse across all studies, an unfortunate pattern that is present throughout much of medical literature to date and must be addressed moving forward given the plans to send both men and women on future long-duration space missions. As noted above, perhaps the largest limitation is the extraordinarily small sample size, which significantly limits the ability to use the robust statistical methods typically applied to this type of research. While a definitive interpretation of the current data is not currently possible due to these study limitations, the body of work to date represents a Herculean scientific effort and has proven to be exceedingly important to our developing understanding of the impact of spaceflight on the brain. These results have helped to inform and refine further experimental planning, including prospective studies with uniform neuroimaging testing across all crewmembers that are currently underway^16^. As highlighted by Roberts et al.,^66^ the development of standardized protocols for pre- and post-flight studies across nations and space agencies also represents an opportunity to optimize the power and validity of future neuroimaging studies.

## Conclusions

This review summarizes the current findings of neuroimaging-evaluated changes to the brain after spaceflight, and includes comparison to data from spaceflight analog studies. We have also reviewed the evidence indicating that mission duration is a key factor in spaceflight-associated brain alterations, and have highlighted potential sources of CNS-related risk to crew performance during spaceflight. A summary of the CNS regions with altered structure or functional connectivity, their physiologic function, and the potential consequences of pathologic changes within these brain regions is given in Supplementary Table 1.

**Fig. 2:**
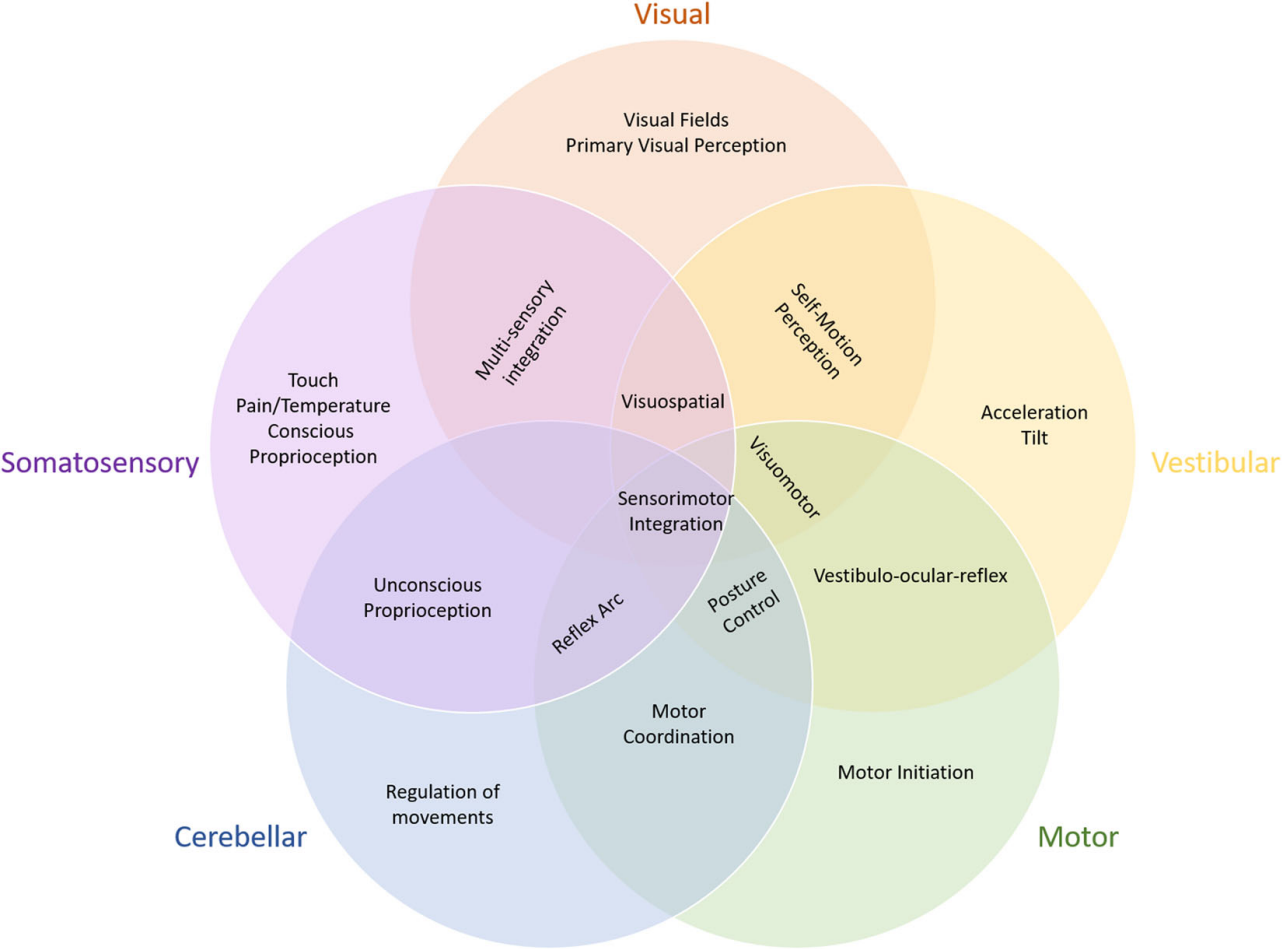
CNS regions impacted during spaceflight. A summary figure illustrating the CNS regions believed to be impacted during spaceflight, including both primary CNS regions and the integration of those regions during higher-level functionining.

In conclusion, current data suggest that multiple CNS regions are impacted during spaceflight, resulting in structural and functional changes within both the primary regions and in the integration of those regions during higher-level functions (Fig. 2). These changes likely result from the combinatorial effects of multiple spaceflight-associated stressors. However, our review of the literature indicates that microgravity itself may represent the most acutely significant stressor to the CNS during spaceflight. While some CNS alterations likely represent beneficial adaptations to the novel environment of space, the impact of gravitational environment transitions and other potential dysregulations on operational performance remains unclear. Further studies are necessary to identify, monitor, and mitigate potentially maladaptive spaceflight-associated neurological changes because even minor alterations in brain structure and function may significantly impact operationally relevant performance and mission success. Monitoring CNS pathology and maladaptation and developing countermeasures, particularly in response to specific mission-phase risk, should be a top priority in human factors research for future long-duration crewed space exploration.

## Supplementary information

Supplementary Material
