# Supplementary material for: A review of alterations to the brain during spaceflight and the potential relevance to crew in long-duration space exploration
Source: NPJ Microgravity. 2021 Feb 16;7:5. doi: 10.1038/s41526-021-00133-z (PMC7887220; doi:10.1038/s41526-021-00133-z)
Supplement: Supplementary file 1 — Supplementary Material [file 41526_2021_133_MOESM1_ESM.pdf]

Supplementary Material

Group	Sequence	Physics	Characteristics	Utility
Spin Echo	T1-Weighted MRI	Longitudinal, spin-lattice relaxation	Low signal intensity of tissue with high water content, high signal intensity of high fat tissue (white matter) [1]	Good anatomical detail, macroscopic overview of tissue[1]
	T2-Weighted MRI	Transverse, spin-spin relaxation	High signal intensity of tissue with high water content (edema, infarction, inflammation), intermediate intensity of high fat tissue (white matter)[1]	Foundational overview of standard brain anatomy, assists in evaluation of brain pathology[1]
Inversion Recovery	Fluid-attenuated inversion recovery	Selective suppression of fluid (CSF) signal	Reduced intensity of CSF signal, high signal intensity of tissue edema/inflammation[2]	Distinguishes subtle white matter abnormalities[2]
Diffusion Weighted	Conventional Diffusion Weighted Imaging (DWI)	Measures the diffusion of water molecules	Increased signal intensity in areas with restricted diffusion of water[3]	Allows mapping of the diffusion of water within the brain[3]
	Diffusion Tensor Imaging (DTI)	Measures the anisotropic diffusion of water in the brain	Estimation of axonal (white matter) organization within the brain[4]	Assessment of white matter tracts, deformations of white matter secondary to brain pathology
Functional MRI	Blood-oxygen-level-dependent (BOLD) imaging	Measures properties of hemoglobin to assess local changes in blood flow, with correlation to activity	Identification of regions of activity within the brain[5]	Visualization of areas of increased cortical activity during rest or activation[5]

Supplementary Table 1. Overview of MRI sequence types utilized in the studies discussed within this review.

Structure	Function	System	Functional Symptoms
Superior Longitudinal Fasiculus (Right)[6]	Connects the frontal, occipital, parietal, and temporal lobes. Visuospatial attention (goal-directed, stimulus driven) and motor control.[7]	Visual, motor, cognitive, behavioral	Observed: Crewmembers with the largest spaceflight-associated balance disruptions had greater WM structure changes in the SLF[6]<br><br>Potential: Visuospatial cognitive dysfunction (searching, shifting spatial attention, performing mental rotations, detecting patterns)[7]
Inferior Longitudinal Fasiculus[6]	Connects temporal and occipital lobes. Visual modality, object/face/place processing, emotional processing, visual memory.[8]	Visual, cognitive, behavioral	Potential: Visual neglect, visual amnesia, visual hallucinations, prosopagnosia, visual hypo-emotionality[8]
Inferior Occipitofrontal Fasiculus[6]	Connects frontal and occipital/temporal lobes. Hypothesized role in spatial attention and cognition (non-dominant), goal-oriented behavior, social cognition, attention[9]	Visual, cognitive, behavioral	Potential: Reduced verbal fluency, spatial neglect, anosognosia, potential for inability to identify facial emotions[9]
R. posterior thalamic radiations[10]	Posterior radiations connect thalamus and occipital cortex, contain optic radiations.[11]	Visual	Potential: Visual pathway deficits[11]
Calcarine, middle occipital, inferior occipital gyri[10]	Location of the primary visual cortex and secondary visual association cortex[12]	Visual, cognitive	Potential: Visual hallucinations, visual field cuts[12]
Right fusiform gyrus[10]	Facial and word recognition, within-category identification[13]	Visual, cognitive, behavioral	Potential: Prosopagnosia, dyslexia[13]
Inferior cerebellar peduncle[6]	Connects the cerebellum to the spinal cord and medulla. Carries input from vestibular receptors (afferent and efferent) to help with posture, balance, coordination.[14]	Cerebellar, vestibular	Potential: Vertigo and imbalance mimicking a peripheral vestibular issue, ocular tilt, spontaneous nystagmus[14]
Middle cerebellar peduncle[6]	Afferent pathway from pons to cerebellum. Controls the initiation, planning and timing of volitional motor activity[15]	Cerebellar, motor	Potential: Cerebellar ataxia (limb, gait, speech)[15]
Corticospinal Tract[6]	Efferent motor information from the cerebral cortex to the body[16]	Motor	Potential: Central motor weakness[16]
White matter underlying primary motor and sensory cortices[6]	Afferent and efferent motor and sensory information, sensory integration[17,18]	Motor, somatosensory, vestibular, cognitive	Potential: Impairment in voluntary movement, sensory ataxia, altered sensory perception of somatosensory input and vestibular equilibrium[17,18]
Inferior and posterior parietal lobe[6]	Body spatial representation, central vestibular and proprioceptive processing, vertical upright perception[19]	Somatosensory, vestibular, cognitive	Potential: Sensorimotor deficits, including deficits in perception and spatial memory, inaccurate reaching and grasping, inattention, deficits in trunk and limb orientation[19,20]
Thalamus[10]	Relays information between cortex and subcortical areas.[21]	Visual, somatosensory, cognitive, behavioral	Potential: Neuropsychological disturbances, motor weakness or sensory loss, deficits in episodic memory, executive function, processing speed, directed attention, working memory[21]
Frontal poles[22]	Higher order cognition, emotion, goal directed behavior, task prioritization[23]	Cognitive, behavioral	Potential: Loss of empathy, difficulty re-prioritizing tasks[23]

Temporal Poles[22]	Semantic naming, facial recognition, integration of emotion with sensory information.[24]	Visual, cognitive, behavioral	Potential: Social withdrawal, failure to produce appropriate social signals or recognize them, self-centered, loss of empathy, unstable mood states.[24]
Supplementary Motor Region[25,26]	Self-initiated movement, stimulus-cued movement, speech production, temporal triggering.[27,28]	Motor, visual, cognitive, behavioral	Potential: Akinesia, speech deficits, executive working memory deficits[27,28]
Premotor Region[25]	Integration of visuospatial information for movement planning, both external and internal memory-based[29]	Visual, motor, cognitive, behavioral	Potential: Impaired performance of visually and verbally cued tasks, difficulty initiating memory cued tasks, motor apraxia[30]
Primary Sensorimotor Region[25]	Integration of sensory and motor skills needed for skilled movement[18]	Motor, somatosensory	Potential: Impaired cross motor function and reflexes, impaired fine motor skills, disrupted learning of new motor tasks[18]
Left Caudate Nucleus[25]	Voluntary movement, postural control, cognitive planning of adaptive goal-directed behavior.[31]	Motor, somatosensory, cognitive	Observed: Decrement in postural control on Recovery from Fall/Stand Test, Dynamic Postural Stability Test.[25]<br><br>Potential: Gait disturbances, slowed walking speed. Impaired visual discrimination, alternation behavior, cognitive-driven strategy switching.[31-33]
Lower Extremity Primary Motor Area/Mid-Cingulate[25]	Motor execution. Processing of internal/external states (emotional states) and translation into motor commands.[34]	Motor, cognitive, behavioral	Potential: Impaired movement[34]

Supplementary Table 2. Correlation between the structure/connectivity alterations seen with neuroimaging after spaceflight, their relationship to major CNS pathways, and the predicted sensorimotor/cognitive/behavioral symptoms associated with deficits within the pathway.

References

1 DG, M. *MRI Principles*. (Saunders, 2004).
2 Okuda, T. *et al.* Brain lesions: when should fluid-attenuated inversion-recovery sequences be used in MR evaluation? *Radiology* **212**, 793-798, doi:10.1148/radiology.212.3.r99se07793 (1999).
3 Hagmann, P. *et al.* Understanding diffusion MR imaging techniques: from scalar diffusion-weighted imaging to diffusion tensor imaging and beyond. *Radiographics* **26 Suppl 1**, S205-223, doi:10.1148/rg.26si065510 (2006).
4 Jellison, B. J. *et al.* Diffusion tensor imaging of cerebral white matter: a pictorial review of physics, fiber tract anatomy, and tumor imaging patterns. *AJNR Am J Neuroradiol* **25**, 356-369 (2004).
5 Khanna, N., Altmeyer, W., Zhuo, J. & Steven, A. Functional Neuroimaging: Fundamental Principles and Clinical Applications. *Neuroradiol J* **28**, 87-96, doi:10.1177/1971400915576311 (2015).
6 Lee, J. K. *et al.* Spaceflight-Associated Brain White Matter Microstructural Changes and Intracranial Fluid Redistribution. *JAMA Neurol* **76**, 412-419, doi:10.1001/jamaneurol.2018.4882 (2019).
7 Conner, A. K. *et al.* A Connectomic Atlas of the Human Cerebrum-Chapter 10: Tractographic Description of the Superior Longitudinal Fasciculus. *Oper Neurosurg (Hagerstown)* **15**, S407-S422, doi:10.1093/ons/opy264 (2018).
8 Sali, G. *et al.* A Connectomic Atlas of the Human Cerebrum-Chapter 11: Tractographic Description of the Inferior Longitudinal Fasciculus. *Oper Neurosurg (Hagerstown)* **15**, S423-S428, doi:10.1093/ons/opy265 (2018).
9 Conner, A. K. *et al.* A Connectomic Atlas of the Human Cerebrum-Chapter 13: Tractographic Description of the Inferior Fronto-Occipital Fasciculus. *Oper Neurosurg (Hagerstown)* **15**, S436-S443, doi:10.1093/ons/opy267 (2018).
10 Riascos, R. F. *et al.* Longitudinal Analysis of Quantitative Brain MRI in Astronauts Following Microgravity Exposure. *J Neuroimaging* **29**, 323-330, doi:10.1111/jon.12609 (2019).
11 Prasad, S. & Galetta, S. L. Anatomy and physiology of the afferent visual system. *Handb Clin Neurol* **102**, 3-19, doi:10.1016/B978-0-444-52903-9.00007-8 (2011).
12 Baker, C. M. *et al.* A Connectomic Atlas of the Human Cerebrum-Chapter 9: The Occipital Lobe. *Oper Neurosurg (Hagerstown)* **15**, S372-S406, doi:10.1093/ons/opy263 (2018).
13 Weiner, K. S. & Zilles, K. The anatomical and functional specialization of the fusiform gyrus. *Neuropsychologia* **83**, 48-62, doi:10.1016/j.neuropsychologia.2015.06.033 (2016).
14 Choi, J. H. *et al.* Inferior cerebellar peduncular lesion causes a distinct vestibular syndrome. *Eur J Neurol* **22**, 1062-1067, doi:10.1111/ene.12705 (2015).
15 Morales, H. & Tomsick, T. Middle cerebellar peduncles: Magnetic resonance imaging and pathophysiologic correlate. *World J Radiol* **7**, 438-447, doi:10.4329/wjr.v7.i12.438 (2015).
16 Van Wittenberghe, I. C. & Peterson, D. C. in *StatPearls* (2020).
17 Bashir, S. *et al.* Short-term effects of unilateral lesion of the primary motor cortex (M1) on ipsilesional hand dexterity in adult macaque monkeys. *Brain Struct Funct* **217**, 63-79, doi:10.1007/s00429-011-0327-8 (2012).
18 Borich, M. R., Brodie, S. M., Gray, W. A., Ionta, S. & Boyd, L. A. Understanding the role of the primary somatosensory cortex: Opportunities for rehabilitation. *Neuropsychologia* **79**, 246-255, doi:10.1016/j.neuropsychologia.2015.07.007 (2015).
19 Freund, H. J. Somatosensory and motor disturbances in patients with parietal lobe lesions. *Adv Neurol* **93**, 179-193 (2003).

20 Darling, W. G., Bartelt, R. & Rizzo, M. Unilateral posterior parietal lobe lesions disrupt kinaesthetic representation of forearm orientation. *J Neurol Neurosurg Psychiatry* **75**, 428-435, doi:10.1136/jnnp.2003.026138 (2004).

21 Fama, R. & Sullivan, E. V. Thalamic structures and associated cognitive functions: Relations with age and aging. *Neurosci Biobehav Rev* **54**, 29-37, doi:10.1016/j.neubiorev.2015.03.008 (2015).

22 Koppelmans, V., Bloomberg, J. J., Mulavara, A. P. & Seidler, R. D. Brain structural plasticity with spaceflight. *NPJ Microgravity* **2**, 2, doi:10.1038/s41526-016-0001-9 (2016).

23 Pirau, L. & Lui, F. in *StatPearls* (2020).

24 Olson, I. R., Plotzker, A. & Ezzyat, Y. The Enigmatic temporal pole: a review of findings on social and emotional processing. *Brain* **130**, 1718-1731, doi:10.1093/brain/awm052 (2007).

25 Roberts, D. R. *et al.* Prolonged Microgravity Affects Human Brain Structure and Function. *AJNR Am J Neuroradiol*, doi:10.3174/ajnr.A6249 (2019).

26 Hupfeld, K. E. *et al.* The Impact of 6 and 12 Months in Space on Human Brain Structure and Intracranial Fluid Shifts. *Cereb Cortex Commun* **1**, tgaa023, doi:10.1093/texcom/tgaa023 (2020).

27 Lima, C. F., Krishnan, S. & Scott, S. K. Roles of Supplementary Motor Areas in Auditory Processing and Auditory Imagery. *Trends Neurosci* **39**, 527-542, doi:10.1016/j.tins.2016.06.003 (2016).

28 Picard, N. & Strick, P. L. Activation of the supplementary motor area (SMA) during performance of visually guided movements. *Cereb Cortex* **13**, 977-986, doi:10.1093/cercor/13.9.977 (2003).

29 Mazurek, K. A. & Schieber, M. H. Injecting Instructions into Premotor Cortex. *Neuron* **96**, 1282-1289 e1284, doi:10.1016/j.neuron.2017.11.006 (2017).

30 Purves D, A. G., Fitzpatrick D, et al., editors. in *Neuroscience. 2nd Edition.* (Sinaeur Associates, 2001).

31 Grahn, J. A., Parkinson, J. A. & Owen, A. M. The cognitive functions of the caudate nucleus. *Prog Neurobiol* **86**, 141-155, doi:10.1016/j.pneurobio.2008.09.004 (2008).

32 Lee, K. B. *et al.* Brain lesions affecting gait recovery in stroke patients. *Brain Behav* **7**, e00868, doi:10.1002/brb3.868 (2017).

33 Pasquini, J. *et al.* Clinical implications of early caudate dysfunction in Parkinson's disease. *J Neurol Neurosurg Psychiatry* **90**, 1098-1104, doi:10.1136/jnnp-2018-320157 (2019).

34 Strick, P. L., Dum, R. P. & Picard, N. Motor areas on the medial wall of the hemisphere. *Novartis Found Symp* **218**, 64-75; discussion 75-80, 104-108 (1998).
